# Diagnostic value of combined detection of serum NLR and CRP for migraine patients in the attack stage: A prospective study

**DOI:** 10.5937/jomb0-57701

**Published:** 2026-01-06

**Authors:** Yanluan Wan, Guanglan Liu, Chunfu Tao, Xiujuan Yuan, Haijian Zheng

**Affiliations:** 1 Ganyu District People's Hospital, Department of Neurology, Lianyungang, Jiangsu

**Keywords:** migraine disorders, C-reactive protein, biomarkers, neutrophils, lymphocytes, migrena, C-reaktivni protein, biomarkeri, neutrofili, limfociti

## Abstract

**Background:**

We attempted to clarify the diagnostic value of combined detection of neutrophil-to-lymphocyte ratio (NLR) and serum C-reactive protein (CRP) for migraine patients in the attack stage.

**Methods:**

A total of 50 migraine patients in the attack stage undergoing treatment in our hospital from June 2023 to June 2024 were chosen as the observation group. Additionally, 50 healthy individuals undergoing physical examination in our hospital were chosen as the control group. We adopted questionnaires to obtain detailed demographic data, medical history, and disease characteristics of patients. The patients and healthy examinees received routine blood tests without prior medication within 3 hours after admission. The absolute values of neutrophils (N) and lymphocytes (L) were obtained, followed by calculation of the neutrophil-to-lymphocyte ratio (NLR). The serum C-reactive protein (CRP) level was measured using immuno-nephelometry. The diagnostic value of NLR and serum CRP for migraine was analysed using the receiver operating characteristic (ROC) curve.

**Results:**

The serum CRP and NLR levels were significantly higher in the observation group compared with the control group (P&lt; 0.05). In the observation group, serum CRP and NLR levels in patients with migraine with aura were comparable to those in patients without aura, with no significant difference (P&gt; 0.05). Similarly, differences in serum CRP and NLR levels between patients with frequent migraine attacks and those with infrequent attacks were not statistically significant (P&gt; 0.05). Serum CRP or NLR alone could be used to diagnose migraine patients in the attack stage, and there was no significant difference between them in diagnostic accuracy (P= 0.633). However, combined detection of serum CRP and NLR showed a significantly higher diagnostic value than either marker alone.

**Conclusions:**

The inflammatory biomarkers serum CRP and NLR were markedly elevated in migraine patients during the attack stage. The combination of serum CRP and NLR has diagnostic value in identifying migraine attacks.

## Abstract (sr)

**Uvod:**

Cilj ovog istraživanja bio je da se utvrdi dijagnostička vrednost kombinovanog određivanja odnosa neutrofila i limfocita (NLR) i serumskog C-reaktivnog proteina (CRP) kod pacijenata sa migrenom u fazi napada.

**Metode:**

U ispitivanje je uključeno ukupno 50 pacijenata sa migrenom u fazi napada, koji su se lečili u našoj ustanovi u periodu od juna 2023. do juna 2024. godine, i oni su činili posmatranu grupu. Kontrolnu grupu je činilo 50 zdravih osoba koje su obavile sistematski pregled u istoj ustanovi. Korišćeni su upitnici za prikupljanje detaljnih demografskih podataka, medicinske istorije i karakteristika bolesti kod pacijenata. Svi ispitanici su, bez prethodne terapije, u roku od 3 sata nakon prijema, podvrgnuti rutinskoj analizi krvi. Dobijene su apsolutne vrednosti neutrofila (N) i limfocita (L), a zatim je izračunat odnos neutrofila i limfocita (NLR). Nivo serumskog C-reaktivnog proteina (CRP) meren je imunonefelometrijom. Dijagnostička vrednost NLR i CRP za migrenu analizirana je pomoću ROC krive.

**Rezultati:**

Nivoi serumskog CRP i NLR bili su značajno viši u posmatranoj grupi u poređenju sa kontrolnom grupom (P&lt; 0,05). U posmatranoj grupi, nivoi CRP i NLR kod pacijenata sa migrenom sa aurom se nisu značajno razlikovali od onih kod pacijenata bez aure (P&gt; 0,05). Takođe, razlike u nivoima CRP i NLR između pacijenata sa čestim i ređim napadima migrene nisu bile statistički značajne (P&gt; 0,05). I CRP i NLR, pojedinačno, mogli su se koristiti za dijagnostikovanje migrene u fazi napada, bez značajne razlike u tačnosti dijagnoze (P= 0,633). Međutim, kombinovano određivanje serumskog CRP i NLR pokazalo je znatno veću dijagnostičku vrednost u poređenju sa pojedinačnim parametrima.

**Zaključak:**

Inflamatorni biomarkeri, serumski CRP i NLR,su značajno povišeni kod pacijenata sa migrenom tokomnapada. Njihova kombinacija ima dijagnostički značaj uidentifikaciji napada migrene.Kljucne reci: migrena, C-rea

## Introduction

Migraine is a common primary headache, with a lifelong prevalence rate of 10%-20% [1] [2], and has a genetic tendency [3], which is listed as the seventh disabling disease in the world [4]. Migraine lasted and recurred, and patients' major symptoms were nausea, vomiting, headache, accompanied by fear of sound and light to varying degrees, etc. [5]. When migraine is sensitive to sound or light stimulation, it will induce a headache attack or aggravate other symptoms [6]. The pathogenesis of migraine remains elusive, and the following three theories are mainly: Vasogenic theory, cortical diffusion inhibition theory, and trigeminal neurovascular theory [7] [8] [9] [10] [11] [12]. In 1984, Moskowitz discovered that electrical stimulation on the trigeminal ganglion could result in aseptic inflammation of dural vessels and believed that neurogenic meningitis was a vital pathophysiological change of migraine [13] [14]. This suggests that neuroinflammation exerts a crucial role in migraine pathogenesis [15]. Thus, it is of great significance to figure out appropriate inflammatory markers for diagnosing migraine patients in the attack stage.

Serum C-reactive protein (CRP) is an acute-phase reactive protein synthesised by the liver and produced by a series of infection or inflammatory cytokines [16] [17]. Serum CRP concentration has a sensitive response to various inflammatory states [18]. CRP is a highly significant change in the acute stage in many patients. For example, in acute trauma and infection, the blood concentration of CRP can reach 200 times the normal level, and then rapidly decrease to normal during the recovery period of the lesion, and has the effect of activating complement and promoting phagocytosis of granulocytes and macrophages. Recent reports have demonstrated that serum CRP level is of great significance for diagnosing pulmonary infections and coronary heart disease [19] [20] [21]. CRP has been utilised clinically for auxiliary diagnosis or prognosis judgment of coronary atherosclerosis, heart failure, chronic obstructive pulmonary disease, etc. [22] [23] [24]. CRP can reflect the degree of inflammation in acute cerebral infarction patients, and CRP has high specificity and sensitivity in predicting neural function deterioration in the acute stage [25]. It has been revealed that plasma CRP of migraine patients presented an upregulation [26]. In recent years, CRP has been widely used in the diagnosis and treatment of cerebrovascular disease, atherosclerosis, and demyelinating diseases. Nevertheless, CRP role in diagnosing migraine in the attack stage is rarely reported. Additionally, neutrophil to lymphocyte ratio (NLR) is a newly proposed inflammation indicator [27], which can reflect the inflammatory response state of the body. It has been demonstrated that migraine may also have a relation to oxidative stress [28]. When oxidative stress occurs, NLR increases; as a cheap and practical marker, NLR can be easily detected and can predict the occurrence and prognosis of multiple diseases [29]. Nevertheless, the NLR role in diagnosing migraine in the attack stage is rarely reported.

Thus, we attempted to clarify the diagnostic value of the combined detection of neutrophil-to-lymphocyte ratio (NLR) and serum C-reactive protein (CRP) in migraine patients during the attack stage. To the best of our knowledge, this is one of the first prospective studies to evaluate the combined utility of NLR and CRP as inflammatory biomarkers specifically for diagnosing migraine attacks, potentially offering a cost-effective and straightforward adjunct to clinical assessment.

## Materials and methods

### General data

A total of 50 migraine patients in the attack stage undergoing treatment in our hospital from June 2023 to June 2024 were chosen as the observation group, including 23 males and 27 females, aged 21-60 years, with a mean age of 42.4±14.9 years. Additionally, 50 healthy people undergoing physical examination in our hospital were chosen as the control group, including 18 males and 32 females, aged 18-59 years, with a mean age of 41.3±18.8 years. General data (age and sex) presented no difference between the two groups (P>0.05), which showed comparability. Inclusion criteria of observation group:1) Conforming to diagnostic criteria for migraine formulated by the International Headache Society [30];2) aged 18-65 years; 3) those with normal vital signs and normal results by neurological examination; 4) patients knew the research content and signed the informed consent, and the ethics committee of our hospital approved research. Inclusion criteria of control group: Those with normal vital signs and normal results by physical examination. Exclusion criteria: 1) Those with other types of non-migraine headaches; 2) those who received migraine prevention or therapy 1 month before research; 3) those with heart, liver and kidney dysfunction, diabetes, rheumatic diseases and malignancies; 4) those who had taken NSAIDs within one week before research; 5) pregnant and breastfeeding women; 6) those with acute infection; 7) any other acute or chronic pain disorders except migraine.

### Data collection

Our research was prospective research, which adopted questionnaires to obtain the demographics, medical history and disease situation of patients in detail. The migraine attack frequency, ache location, type and intensity, and other related symptoms (nausea, vomiting, voice phobia, photophobia, visual impairment, etc.) were recorded. According to the diagnostic criteria of migraine, the presence or absence of migraine aura was judged, and 50 migraine patients were divided into migraine with aura group (n = 21) and migraine without aura group (n=29). According to the International Classification of Headache Disorders, 3rd edition (ICHD-3) [31], patients experiencing 4 migraine attacks per month were classified as the *frequent attack group* (n = 14), while those with <4 attacks per month were assigned to the *infrequent attack group* (n = 36).

### Laboratory tests

The patients and health examinees received routine blood examinations if no medication was taken within three hours after admission. The absolute values of neutrophil (N) and lymphocyte (L) were obtained, followed by calculation of NLR. The 5 mL of venous blood was taken from patients and health examinees, followed by centrifugation at 4000 rpm for 10 min, and then the serum was stored at -80°C in a refrigerator for standby. The serum CRP level was measured through immune nephelometry with a SIEMENS automatic protein analyser. All measurements were completed by two operators who had received technical training and a qualification assessment.

### Statistical analysis

SPSS 22.0 software was utilised for statistical analysis and plotting. The measurement data conformed to normal distribution and were expressed by (x̄±s). The t-test was utilised to compare groups. The diagnostic value of NLR and serum CRP for migraine was analysed through the receiver operating characteristic curve (ROC). P<0.05 indicated statistical significance.

## Results

### CRP and NLR levels between groups

The results showed that the serum CRP and NLR levels were significantly higher in the observation group compared with the control group. The difference in serum CRP levels was statistically significant (P=0.008), and the difference in NLR was also statistically significant (P=0.009) (Table 1).

**Table 1:** Serum CRP and NLR levels in both groups (x̄±s).

Groups	n	CRP (mg/L)	N (x10^9^/L)	L (x10^9^/L)	NLR
Control group	50	5.06±3.51	4.32±0.54	2.54±0.94	1.70±0.57
Observation group	50	10.05±3.58	5.67±0.46	2.24±0.82	2.53±0.56
t		4.928			5.229
P		0.008			0.009

### CRP and NLR in migraine subtypes

Serum CRP and NLR levels were significantly higher in migraine patients during the attack stage (observation group) compared to healthy controls (P=0.008 for CRP; P=0.009 for NLR) (Table 1).

Within the observation group, serum CRP levels were not significantly different between patients with migraine with aura and those without aura (P=0.243). Likewise, NLR levels did not differ significantly between these subgroups (P=0.405) (Table 2).

**Table 2:** Serum CRP and NLR levels in migraine patients with and without aura (x̄±s).

Groups	n	CRP (mg/L)	N (x10^9^/L)	L (x10^9^/L)	NLR
Migraine with aura group	21	11.80±1.96	6.13±0.23	2.28±0.84	2.69±0.27
Migraine without aura group	29	11.10±2.05	5.33±0.29	2.13±0.92	2.62±0.31
t		0.945			0.719
P		0.243			0.405

Similarly, no statistically significant differences were observed in CRP (P=0.123) or NLR (P=0.068) levels between patients with frequent attacks and those with infrequent attacks (Table 3).

**Table 3:** Serum CRP and NLR levels in patients with frequent and infrequent migraine attacks (x̄±s).

Groups	n	CRP (mg/L)	N (x10^9^/L)	L (x10^9^/L)	NLR
Frequent migraine attack group	14	12.46±2.40	6.48±0.23	2.39±0.83	2.71±0.28
Infrequent migraine attack group	36	11.35±2.10	5.90±0.24	2.31±0.90	2.55±0.27
t		1.241			1.57
P		0.123			0.068

### Diagnostic value of CRP or NLR alone

The ROC curve analysis demonstrated that serum CRP could be used to diagnose migraine patients in the attack stage, with a cutoff value of 5.67 mg/L, sensitivity of 92.00%, and specificity of 82.00%. The area under the curve (AUC) was 0.833, with a 95% confidence interval (CI) of 0.745-0.921 and a P-value of 0.006.

Similarly, NLR had a cutoff value of 2.245, with sensitivity of 80.00% and specificity of 84.00%; the AUC was 0.848, with a 95% CI of 0.769-0.927 and a P-value of 0.007.

**Figure 1:**
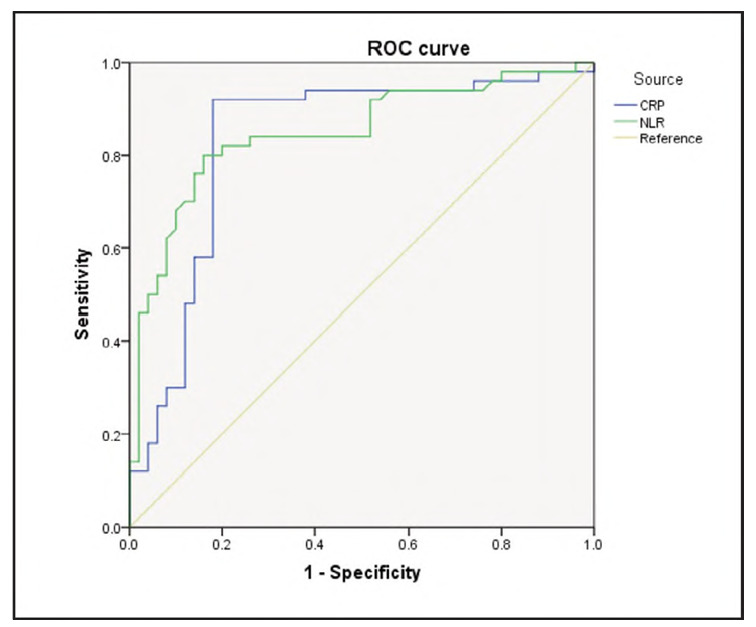
Diagnostic value of serum CRP or NLR for migraine analysed by ROC.

There was no significant difference between the diagnostic values of serum CRP and NLR alone (P=0.633) (Figure 1).

### Combined diagnostic value of CRP and NLR for migraine patients in the attack stage

**Figure 2:**
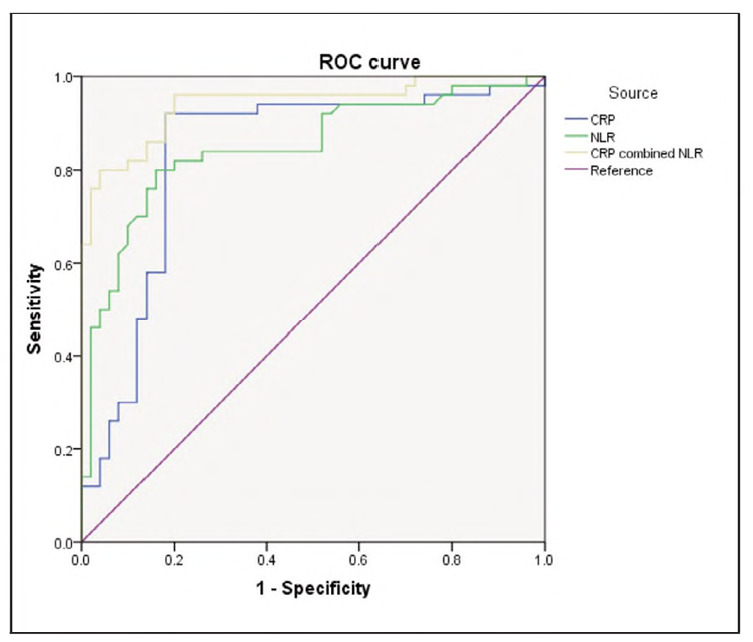
Diagnostic value of combined detection of serum CRP or NLR for migraine analysed by ROC.

Combined detection of serum CRP and NLR showed high diagnostic accuracy for migraine in the attack stage, with a sensitivity of 95.67%, specificity of 97.23%, and an AUC of 0.941 (95% CI: 0.895-0.987, P<0.001). The diagnostic performance of the combined markers was significantly better than that of CRP alone (P = 0.045) and NLR alone (P=0.009) (Figure 2).

## Discussion

The present study aimed to evaluate the diagnostic value of serum CRP and NLR in patients experiencing migraine during the attack stage. Our findings revealed that both CRP and NLR levels were significantly elevated in migraine patients compared to healthy controls. Specifically, serum CRP levels in the observation group averaged 10.05±3.58 mg/L, which was considerably higher than the 5.06±3.51 mg/L observed in the control group (P=0.008). Similarly, the NLR was elevated in migraine patients (2.53±0.56) compared to controls (1.70±0.57; P=0.009). However, no statistically significant differences in CRP or NLR were observed between migraine subtypes (with aura vs. without aura; frequent vs. infrequent attacks). ROC analysis demonstrated that CRP and NLR had good sensitivity and specificity when used independently for diagnosis. Notably, the combination of CRP and NLR yielded even higher diagnostic performance, indicating that these biomarkers may be more effective when used together.

The role of inflammation in migraine has been increasingly emphasised in recent years, particularly through the lens of the trigemino-vascular theory and neurogenic inflammation hypothesis. When trigeminal nerve fibres are activated, neuropeptides such as substance P (SP), calcitonin gene-related peptide (CGRP), and neurokinin A (NKA) are released, leading to vasodilation and plasma protein extravasation - hallmarks of sterile inflammation within the meninges [32] [33]. These mechanisms support the notion that systemic inflammatory biomarkers, such as CRP and NLR, could reflect migraine activity, especially during acute attacks.

CRP is a classical acute-phase reactant produced by hepatocytes in response to interleukin-6 (IL-6) and other pro-inflammatory cytokines [34]. It is widely recognised as a sensitive marker for detecting early and mild inflammation [35]. In this study, serum CRP levels were significantly higher in migraine patients during the attack stage. This aligns with findings from Tanik et al., who reported elevated high-sensitivity CRP (hs-CRP) levels in migraine patients compared to controls. However, differences between migraines with aura and those without aura were not statistically significant - an observation that mirrors our results [36]. Similarly, Vanmolkot and de Hoon [26] found increased CRP levels in young adult migraine sufferers, suggesting that CRP may be a consistent indicator of systemic inflammation in this patient population. Conversely, Gudmundsson et al. [37] found no significant difference in CRP levels between migraineurs and non-migraineurs in a large population-based study and older women with migraine without aura had lower CRP levels than non- migraineurs and those with aura, suggesting no consistent link between CRP and migraine. In our study, CRP levels did not significantly differ across migraine subtypes or attack frequency categories, indicating that while CRP is a reliable marker for detecting migraine in general, it may not be suitable for distinguishing between clinical subtypes.

NLR has emerged as a novel, inexpensive, and easily accessible marker for systemic inflammation and immune stress. Unlike absolute leukocyte counts, NLR reflects a balance between the innate (neutrophil-mediated) and adaptive (lymphocyte-mediated) immune responses [38]. Elevation in NLR may thus signal a pro-inflammatory state even when total white blood cell counts remain within normal limits [39] [40]. This study found a significant increase in NLR among migraine patients compared to healthy individuals, which is consistent with previous findings. Karabulut et al. [41] demonstrated elevated NLR values during migraine attacks in comparison to both healthy controls and interictal migraine states. Another study by Lee et al. [42] analysed 10 years of clinical data and found that peripheral inflammatory markers, especially NLR and neutrophil-to-monocyte ratio (NMR), were elevated during migraine attacks. While these markers poorly differentiated migraine from non-migraine headaches, they may still support the role of inflammation in migraine diagnosis. Our findings not only confirm the utility of NLR in identifying acute migraine episodes but also suggest that, like CRP, NLR lacks discriminatory power between aura-related subtypes and attack frequencies.

Although both CRP and NLR demonstrated good diagnostic performance independently, the most promising finding of this study is the significantly higher diagnostic value when both biomarkers are combined. The combined model yielded a sensitivity of 95.67% and a specificity of 97.23%. These results suggest that integrating multiple inflammatory biomarkers may improve diagnostic accuracy, potentially compensating for the nonspecific nature of each marker individually. This aligns with a growing body of evidence in other neurological and inflammatory disorders, where combined biomarker panels have demonstrated superior diagnostic and prognostic utility compared to single markers [43] [44] [45].

From a clinical perspective, the potential to use CRP and NLR as rapid, cost-effective adjuncts to clinical evaluation in the emergency department or outpatient setting is highly valuable. Because both markers are part of routine blood panels, they can be easily implemented without significant additional costs or infrastructure. However, one critical factor that must be addressed in future studies is the timing of sample collection relative to the onset of migraine symptoms. Inflammatory markers may fluctuate significantly depending on whether the patient is in the early, peak, or resolving phase of an attack. We recommend that future studies consider standardising blood collection to within 1 hour of symptom onset or evaluating dynamic changes in these markers across different attack stages.

A growing number of studies have attempted to assess systemic inflammatory markers in migraine, though findings remain somewhat inconsistent. For example, Togha et al. [46] reported elevated oxidative stress and inflammation markers in migraine patients, including increased levels of nitric oxide and decreased antioxidant capacity. Similarly, Sudershan et al. highlighted the importance of inflammatory cytokines such as TNF-α and IL-6 in chronic migraine pathogenesis [47]. While these studies do not directly assess CRP or NLR, they support the hypothesis that systemic inflammation plays a central role in migraine. Our study contributes to this literature by focusing on accessible and standardised blood markers and confirming their diagnostic value in a prospective clinical setting.

Despite the strengths of our research, including a prospective design and matched control group, some limitations must be acknowledged. First, the sample size was relatively small, and subgroup analyses (e.g., migraine with vs. without aura) may have been underpowered to detect subtle differences. Second, our study did not include follow-up blood samples to track changes in CRP and NLR levels across different migraine phases. This limits our ability to assess the dynamic inflammatory response over time. Third, although we used the ICHD-3 to define frequent and infrequent migraine attacks, we did not explore other clinical features such as duration, intensity, or associated symptoms in relation to inflammatory markers. Finally, both CRP and NLR are nonspecific indicators and may be elevated in a wide range of other conditions, including infections, autoimmune disorders, and metabolic syndromes. Although we excluded patients with known comorbidities, subclinical conditions could not be entirely ruled out.

In conclusion, our study demonstrates that both serum CRP and NLR are significantly elevated in migraine patients during the attack stage. While neither marker alone was able to distinguish between migraine subtypes or attack frequency, their combined use significantly improved diagnostic performance. These findings suggest that CRP and NLR may serve as valuable adjuncts to clinical evaluation in diagnosing acute migraine. Future studies with larger sample sizes and longitudinal follow-up are needed to validate these findings and to explore the utility of these markers in monitoring disease progression and treatment response.

## Dodatak

### Acknowledgments

The authors would like to thank the staff of Ganyu District People's Hospital for their assistance and support throughout the study.

### Funding

This work was supported by the Lianyungang Municipal Health Commission, General Project (No. 202344).

### Ethical consideration

This study was conducted in accordance with the Declaration of Helsinki. Ethical approval was obtained from the Ethics Committee of Ganyu District People's Hospital. Informed consent was obtained from all participants before enrollment.

### Authors' contribution

Yanluan Wan and Guanglan Liu contributed to data collection and patient recruitment. Chunfu Tao and Xiujuan Yuan performed the laboratory testing and data analysis. Haijian Zheng designed the study, supervised the project, and revised the manuscript. All authors read and approved the final manuscript.

### Conflict of interest statement

All the authors declare that they have no conflict of interest in this work.
